# Promoter hypermethylation and comprehensive regulation of ncRNA lead to the down-regulation of ZNF880, providing a new insight for the therapeutics and research of colorectal cancer

**DOI:** 10.1186/s12920-023-01571-2

**Published:** 2023-06-27

**Authors:** Xiangqian Dong, Yinghui Zhang, Yang Sun, Qiong Nan, Maojuan Li, Lanqing Ma, Lei Zhang, Juan Luo, Yating Qi, Yinglei Miao

**Affiliations:** 1 Department of Gastroenterology, The First Affiliated Hospital of Kunming Medical University, NO.295 Xichang Road, Kunming, 650032 P.R. China; 2 Yunnan Province Clinical Research Center for Digestive Diseases, Kunming, 650032 China; 3 Department of Gastroenterology, Affiliated Hospital of Yunnan University, Kunming, 650021 China

**Keywords:** KZFPs, Colorectal carcinoma, *ZNF880*, ceRNA

## Abstract

The human genome encodes more than 350 kinds of Krüppel-associated box (KRAB) domain-containing zinc-finger proteins (KZFPs), KRAB-type ZNF transcription factor family (KZNF) plays a vital role in gene regulatory networks. The KZNF family members include a large number of highly homologous genes, gene subtypes and pseudogenes, and their expression has a high degree of tissue specificity and precision. Due to the high complexity of its regulatory network, the KZNF gene family has not been researched in sufficient, and the role of its members in the occurrence of cancer is mostly unexplored. In this study, *ZNF880* was significantly associated with overall survival (OS) and disease-free survival (DFS) in colorectal carcinoma (CRC) patients. Low *ZNF880* expression resulted in shorter OS and DFS. Combined with Colon adenocarcinoma (COAD) and Rectum adenocarcinoma (READ) data collection in the TCGA database, we found that *ZNF880* was significantly down-regulated in CRC. Further analysis of the sequence variation of *ZNF880* in CRC showed that ZNF880 accumulated a large number of SNV in the C2H2 domain and KRAB domain, while promoter region of *ZNF880* also showed high methylation in COAD and READ. Combined with the Cbioportal and TIMER databases, the expression of mutant *ZNF880* was significantly lower in COAD compared to the wild type. Simultaneously, the lncRNA-miRNA-ZNF880 ceRNA regulatory network was constructed through co-expression and miRNAs target gene prediction, demonstrating the precision of the *ZNF880* regulatory network. In addition, the decreased expression of ZNF880 caused the significant immune infiltration decreases of CD8 + cells in COAD. In contrast, the immune infiltration of CD4 + cells and macrophages in COAD is positively correlated with *ZNF880*. Finally, through protein–protein interaction (PPI) network analysis and transcription factor target gene prediction, we screened out the genes most likely to be related to the function of *ZNF880*. *CENPK, IFNGR2*, *REC8* and *ZBTB17* were identified as the most closely functioning genes with *ZNF880*, which may indicate that *ZNF880* has important links with the formation of cell centromere, tumor immunity, cell cycle and other pathways closely related to the occurrence of CRC. These studies show that the down-regulation of *ZNF880* gene is closely related to CRC, and the targeted change of the expression of its regulatory molecules (miRNA and lncRNA) may be a new perspective for CRC treatment.

**Supplementary Information:**

The online version contains supplementary material available at 10.1186/s12920-023-01571-2.

## Introduction

Colorectal cancer (CRC) is one of the most common cancers and is considered to be the fourth leading cause of cancer-related deaths worldwide [1–3]. Most CRCs originate from non-cancerous lesions and have strong genetic instability [4–6]. The genetic instability of CRC is caused by at least three different mechanisms, including chromosomal instability, CpG island methylation and microsatellite instability [7, 8]. Hypermethylation of CpG islands can trigger the silencing of a series of tumor suppressor genes, thereby making CRC present the characteristics of epigenetic instability [9–11].

KZNFs are the most abundant family of epigenetic inhibitors found in humans [12], and they may be involved in development, metabolism, cell proliferation and carcinogenesis [13]. After KZNFs bind to DNA, KRAB-ZNF triggers transcriptional inhibition by interacting with KAP1 (KRAB-related protein-1) [14, 15]. KAP1 acts as a scaffold for a multimolecular entity comprising the heterochromatin protein 1 (HP1), the H3K9me3-specific histone methyltransferase SETDB1, and the histone deacetylase-containing complex NuRD [16]. These proteins form transcriptional silence by making chromatin form heterochromatin [16, 17]. In addition, KZNFs may also mediate the methylation of DNA sequences adjacent to their binding motifs, thereby triggering silencing of their gene expression [18, 19].

Growing reports indicate that some members of the KZNFs gene family are involved in all aspects of cancer [20, 21]. Among them, ZNF545, ZBKR1, and ZNF307 are considered to have an inhibitory effect on tumorigenesis [22–24]. Due to the hypermethylation of its promoter, the expression of ZNF545 in a variety of malignant tumors is reduced, which leads to its negative control of tumor cell proliferation. ZBKR1 and ZNF307 showed inhibitory activity against cervical cancer and liver cancer, respectively. Interestingly, ZNF224 can perform different functions according to the difference in tumor microenvironment [25]. ZNF224 has been shown to enhance the signal transduction activity of WT1 in chronic myelogenous leukemia cell lines [25]. WT1 is a well-known tumor suppressor gene that controls the expression of genes involved in differentiation, apoptosis and cell cycle progression. However, ZNF224 is used as an oncogene in bladder cancer to promote the rapid growth of tumor cells and the resistance to tumor cell apoptosis. In general, most of the KZNFs family members discovered so far act more as tumor suppressor genes.

In the current research, we aim to explore the possible functions of ZNF880 in CRC by mining the gene expression, regulatory network, epigenetic changes and downstream genes of the KZNFs family gene ZNF880 in CRC. To this end, we used The Cancer Genome Atlas (TCGA) and UALCAN to comprehensively analyze the gene expression variation and differential methylation of the promoter region of ZNF880. Furthermore, through comprehensive analysis of ZNF880 target miRNAs and co-expressed lncRNA, a ceRNA network was constructed. Our analysis comprehensively evaluated the sequence variation, gene expression variation, epigenetic difference, ncRNA regulatory network and the correlation with immune infiltration of ZNF880 in CRC. These analyses have found the hypothetical role of ZNF880 in CRC. ZNF880 has the potential to become a new target for anti-cancer therapy or a biomarker for clinical patient management. In short, these research results initially revealed the function of ZNF880 in CRC, laying the foundation for further experimental verification and functional research.

## Materials and methods

### Data set selection and gene expression differential analysis

In this study, the differential expression of ZNF880 and the correlation analysis between ZNF and other genes used TCGA and GTEx data sets, and the analysis used GEPIA2 (http://gepia2.cancer-pku.cn/#analysis) and UALCAN (http:/ /ualcan.path.uab.edu/analysis.html) online analysis platform. In addition, the differential methylation of the promoter region of ZNF880 in the COAD and READ datasets was analyzed by UALCAN. Subsequently, UALCAN was used to analyze the differential expression of ZNF880 candidate miRNAs in the COAD and READ datasets.

### Candidate miRNA screening of *ZNF880* and construction of ceRNA regulatory network

MiRWalk2.0(http://zmf.umm.uni-heidelberg.de/apps/zmf/mirwalk2/index.html) predicts miRNAs that may target ZNF880. In the analysis, 11 different prediction algorithms (miRDB, PITA, MicroT4, miRMap, RNA22, miRanda, miRNAMap, RNAhybrid, miRBridge, PICTAR2, TargetScan) are used to predict the possible candidate microRNAs of ZNF880, and finally the microRNAs supported by software of more than 8 levels were selected as candidate molecules. The microRNAs of up-regulated in COAD and READ were further screened by UALCAN as possible regulatory molecules of ZNF880. In order to construct the lncRNA-miRNA-ZNF880 ceRNA regulatory network involving ZNF880, miRWalk2.0 was also used to predict the possible lncRNA target genes of the candidate microRNAs. Similarly, the prediction of miRNA-lncRNA is also performed using miRWalk2.0. Five prediction algorithms (miRWalk, miRanda, PITA, RNAhybrid, TargetScan) were used to predict possible lncRNA targets, and lncRNAs supported by all algorithms are finally screened as candidate molecules. LncExpDB was used to further screen the lncRNAs co-expressed with ZNF880(https://ngdc.cncb.ac.cn/ln-cexpdb/interactions).

### ZNF880 protein result structure, single nucleotide variation (SNV) statistics

In this study, AlphaFold was used to predict the protein structure of ZNF880(https://deepmind.com/blog/article/AlphaFold-Using-AI-for-scientific-discovery). The single nucleotide variation (SNV) of ZNF880 in CRC was further analyzed through CBioPortal (https://www.cbioportal.org/). At the same time, the differential expression of wild-type ZNF880 and mutant ZNF880 in CRC was also analyzed through CBioPortal.

### Protein interaction network and *ZNF880* target gene prediction

STRING is an online database for searching interacting genes (https://string-db.org/). In this study, we performed a STRING search for co-expressed genes and constructed a PPI network with an interaction score > 0.4. Furthermore, ENCORI (http://starbase.sysu.edu.cn/panCancer.php) predicted genes that may have binding sites with ZNF880, and the default parameter configuration was used in the analysis. Then, through GeneCards, we screened transcription factors that bind ZNF880 promoter and enhancer regions, and screened transcription factors that might regulate ZNF880.

### Correlation analysis between *ZNF880* and immune infiltration

TIMER is a comprehensive online resource for systematic analysis of immune infiltration of various cancer types (http://timer.cistrome.org/). In this study, we used TIMER to analyze ZNF880 and four types of immune infiltration (CD4 + T cells, CD8 + T cells, neutrophils, macrophages). TISIDB (http://cis.hku.hk/TISIDB/index.php) is an online web integrated repository portal for tumor-immune system interactions. In this study, we conducted TISIDB to determine ZNF880 and the expression of tumor infiltrating lymphocytes (TIL) in human cancers. According to the gene expression profile, the relative abundance of TILs is inferred through gene set variation analysis. The correlation between ZNF880 and TIL is measured by Spearman's test.

### Overall survival (OS) and disease-free survival(DFS) analysis

GEPIA2 is a portal site that integrates TCGA and GTEx data sets. In this study, GEPIA2.0 was used to analyze the Overall survival (OS) and disease-free survival analysis of *ZNF880* in READ and COAD.

### High-ZNF880 group and Low-ZNF880 group division

GSE14333 was selected as the cohort for the potential function verification of ZNF880, and samples with high ZNF880 expression top50 and samples with low top50 expression in GSE14333 were selected as the High-ZNF880 group and Low-ZNF880 group.

### GSEA analysis

Two data sets, oncogenic signature gene sets and cell type signature gene sets, were downloaded from the MsigDB database(http://www.gsea-msigdb.org/gsea/index.jsp). The difference analysis results between the High-ZNF880 group and the Low-ZNF880 group were used as the input file of GSEA, and the GSEA function in the ClusterProfiler package was used for GSEA enrichment analysis, and the pathways with *p* < 0.05 were selected as the significantly enriched pathways. All statistical tests in this study used T-test.

### Protein extraction and Western blot

All tumor and normal samples were collected from the First Affiliated Hospital of Kunming Medical University. Protein extraction and Western blot analysis were performed on colorectal cancer (CRC) tumor tissues and adjacent normal tissues to detect the expression levels of ZNF880, CDK1, and CENPM. Tissue samples were homogenized in RIPA lysis buffer with protease inhibitors, and the total protein concentration was determined using the BCA protein assay kit. Equal amounts of protein were separated by SDS-PAGE and transferred onto nitrocellulose membranes. After blocking with 5% non-fat milk, the membranes were incubated with primary antibodies against ZNF880, CDK1, CENPM, and β-actin overnight at 4 °C. The membranes were then washed and incubated with secondary antibodies for 1 h at room temperature. The protein bands were visualized using enhanced chemiluminescence (ECL) reagent and quantified using ImageJ software. The relative expression levels of ZNF880, CDK1, and CENPM were normalized to the β-actin expression levels.

## Results

### Pan-cancer perspective and the expression pattern of ZNF880 in CRC

**Fig. 1:**
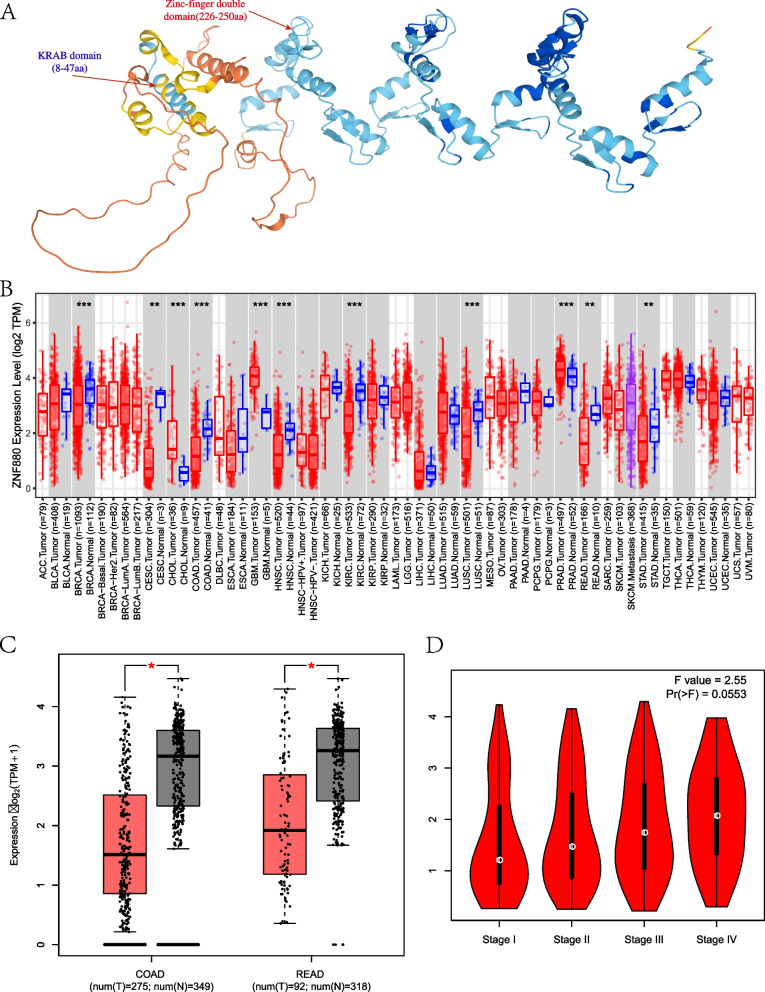
ZNF880 protein structure and its expression in pan-cancer and cancer staging. **A**: The ZNF880 protein structure predicted by AlphaFold, which has typical KRAB and C2H2 domains. **B**: The expression level of ZNF880 in pan-cancer. BLCA, bladder urothelial carcinoma; BRCA, breast invasive carcinoma; CHOL, cholangiocarcinoma; COAD, colon adenocarcinoma; ESCA, esophageal carcinoma; GBM, glioblastoma mutiforme; HNSC, head and neck squamous cell carcinoma; KICH, kidney chromophobe; KIRC, kidney clear cell carcinoma; KIRP, kidney renal papillary cell carcinoma; LIHC, liver hepatocellular carcinoma; LUAD, lung adenocarcinoma; LUSC, lung squamous cell carcinoma; PRAD, prostate adenocarcinoma; READ, rectum adenocarcinoma; STAD, stomach adenocarcinoma; THCA, thyroid carcinoma; UCEC, uterine corpus endometrial carcinoma. **C**: The expression level of ZNF880 in READ and COAD. **D**: The expression level of ZNF880 in CRC cancer staging

ZNF880 belongs to the typical KRAB type ZNF transcription factor family, with typical KRAB and C2H2 domains (Fig. 1A). In order to evaluate the mRNA expression patterns of ZNF880 in different cancer types, we analyzed the differences in the expression of ZNF880 in cancer tissues and normal tissues in the data of more than 23 cancer types. Among them, ZNF880 was down-regulated in more than 7 cancer types, but only up-regulated in two cancers (Fig. 1B). Overall, ZNF880 may exhibit tumor suppressor activity in most cancers. In particular, ZNF880 showed significant down-regulation in READ (num(T) = 275, num(N) = 349) and COAD (num(T = 92), num(N) = 318) (Fig. 1C). Subsequently, the expression of ZNF880 in CRC tumor staging was analyzed, and the results showed that ZNF880 showed an up-regulated expression trend in tumor staging, and overall the expression was significantly down-regulated in the initial stage of tumorigenesis (Fig. 1D).

### Single nucleotide variation and epigenetic changes of ZNF880 in CRC

**Fig. 2:**
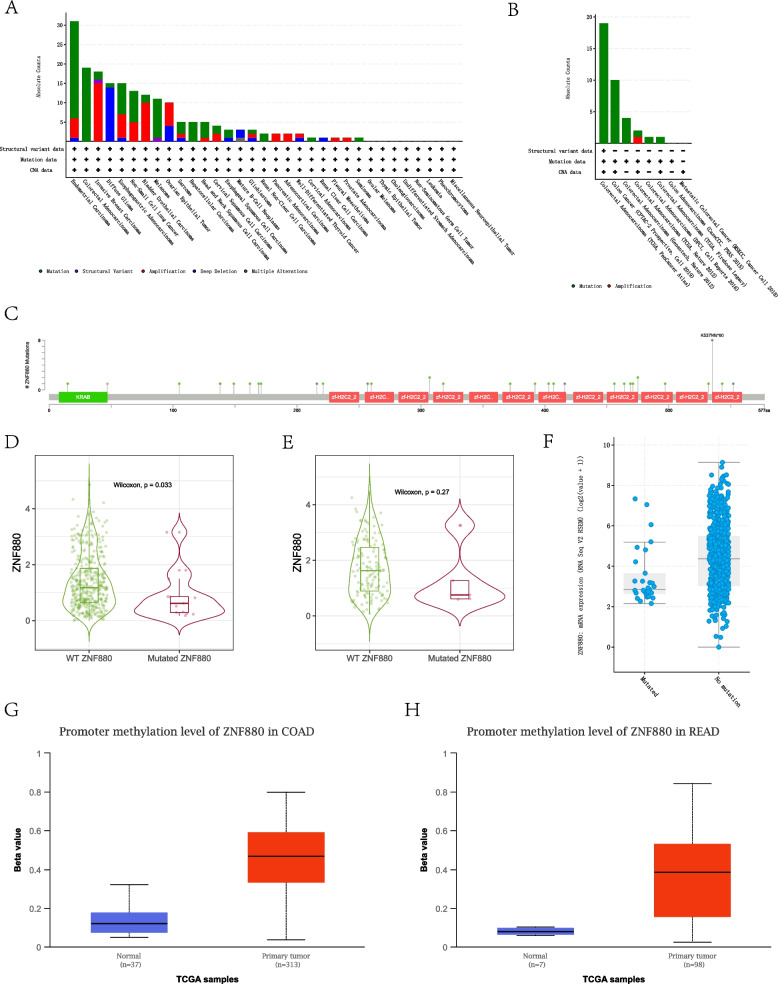
The single nucleotide variation (SNV) statistics and epigenetic changes of ZNF880 in CRC. **A**, **B**: Statistics of single nucleotide variation of ZNF880 in pan-cancer and CRC. **C**: The distribution area of SNV in ZNF880. **D**-**F**: The difference in expression of mutant ZNF880 and wild-type ZNF880 in CRC. G, H: ZNF880 differs in promoter methylation in READ and COAD

SNV, as an important DNA mutation that causes the loss of protein function and affects gene expression, plays a key role in tumorigenesis and diagnosis. In order to evaluate the sequence variation of ZNF880 in CRC, we first counted the variation of ZNF880 in pan-cancer. The results show that ZNF880 has the highest degree of variation in Endometrial Carcinoma and CRC (Fig. 2A). ZNF880 has more than 20 SNVs in CRC, and most of the SNVs are concentrated in KRAB and C2H2 domains (Fig. 2B, C). Comparing the expression levels of wild-type ZNF880 and mutant ZNF880 in COAD and CRC shows that mutant ZNF880 significantly down-regulates expression in COAD (Fig. 2D), while in READ there is a downward trend in expression (Fig. 2E). Verifying the expression level of mutant ZNF880 in more data sets of Cbioportal found that mutant ZNF880 does show a significant expression down-regulation (Fig. 2F). Subsequent analysis of the methylation level of the promoter region of ZNF880 in COAD and READ showed that ZNF880 showed hypermethylation of the promoter region in both COAD and READ(Fig. 2G, H). The hypermethylation of mutant ZNF880 and the promoter region may explain the decrease of ZNF880 expression in CRC to a certain extent.

### ceRNA regulatory network based on ZNF880

**Fig. 3:**
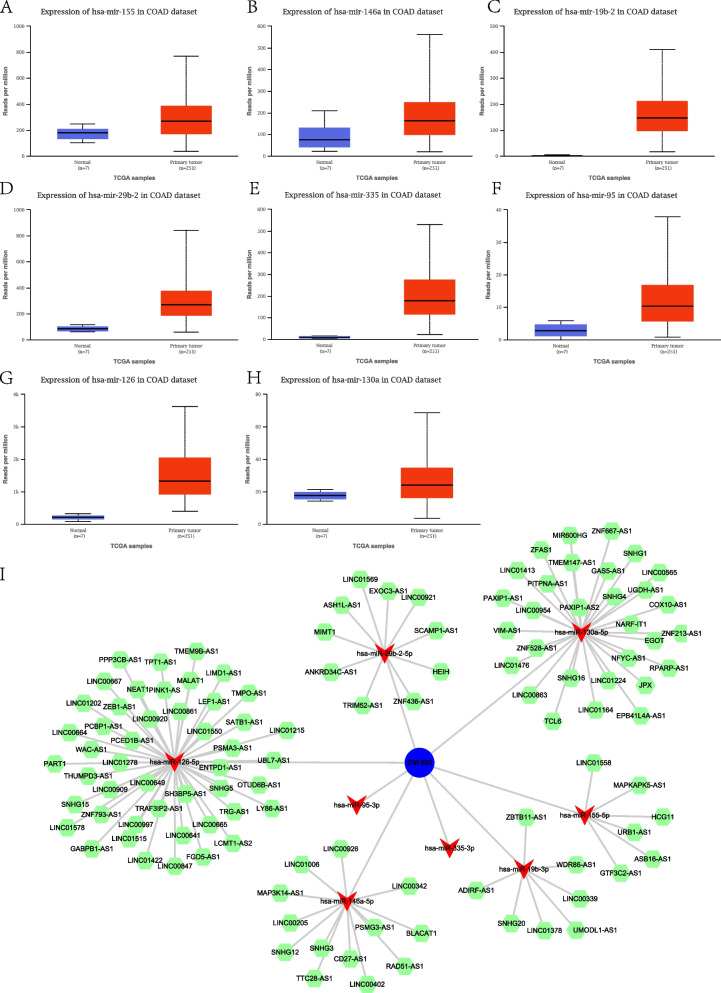
ZNF880 regulates the differential expression of miRNAs and the ceRNA regulatory network. A-H: Eight potential ZNF880 regulate the expression differences of miRNAs in CRC. I: The ceRNA regulatory network of ZNF880. The green represents lncRNA, and the red represents miRNA

The ceRNA (competitive endogenous RNA) regulatory network is considered to be a buffer regulatory mechanism between ncRNA and protein-coding genes. lncRNA can be combined with miRNA, thereby buffering the regulatory effect of miRNA on protein-coding genes. The complex crosstalk of the ceRNA network has been detected in various types of cancers. In order to identify the miRNAs and lncRNAs involved in the ceRNA regulatory network of ZNF880, we first used more than 10 miRNA prediction algorithms to screen the possible binding miRNAs of ZNF880. Then through analysis the expression level of candidate miRNAs in CRC confirmed that there are eight potential ZNF880 regulatory miRNAs. The eight potential regulatory molecules of ZNF880 are hsa-mir-155, hsa-mir-146a, hsa-mir-19b-2, and hsa-mir-29b-2, hsa-mir-335, hsa-mir-95, hsa-mir-126, hsa-mir-130a, they all showed significant up-regulation in CRC (Fig. 3A-H). The up-regulated miRNA may regulate the expression of the target gene ZNF880 through transcription and post-transcriptional levels. In order to further search for lncRNA molecules that may be involved in the regulation of ceRNA, the target lncRNAs of 8 miRNAs were predicted by more than 5 algorithms (Table.S1). Subsequently, by calculating the co-expression of the target lncRNA and ZNF880, the lncRNAs co-expressed with ZNF880 were screened. The significantly co-expressed lncRNAs were screened as potential buffer regulators of ZNF880. Finally, a ZNF880-based ceRNA regulatory network (Fig. 3I) was constructed by integrating miRNAs and lncRNA. Among them, LINC00641, LINC00665, LINC01278, MALAT1, NEAT1, etc. have been shown to significantly down-regulate expression in READ and COAD. These lncRNAs and miRNAs together constitute the fine regulation mechanism of ZNF880.

### Correlation analysis between the expression of ZNF880 and immune cell infiltration in CRC

**Fig. 4:**
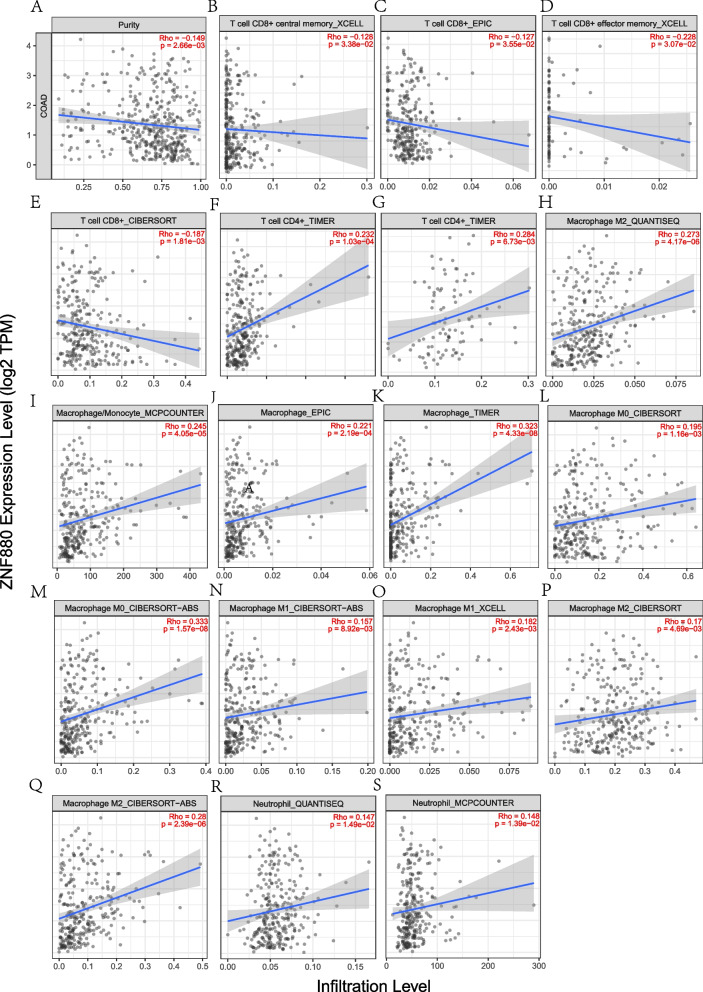
Correlations of ZNF880 expression with immune infiltration level. The figure shows the correlation between ZNF880 and tumor purity, as well as four types of immune cell infiltration. The four types of cells are CD8 + T cells, CD4 + T cells, macrophages and neutrophils. **A**: Correlation between COAD purity and ZNF880 expression. **B**-**E**: Correlation of ZNF880 expression with CD8 + T cell immune infiltration in multiple datasets. **F**, **G**: Correlation between ZNF880 and CD4 + T cell immune infiltration in the TIMER database. **H**-**Q**: Correlation between ZNF880 expression and macrophage immune infiltration in multiple databases. **R**, **S**: Correlation between ZNF880 expression and immune infiltration in neutrophils

We analyzed the correlation between the expression of *ZNF880* in the TIMER database and the four types of tumor infiltrating immune cells, and the expression of *ZNF880* showed a significant negative correlation with tumor (COAD) purity (*r* =  − 0.149, *p* = 2.66e − 03)(Fig. 4A). ZNF880 expression and CD8 + T cell immune infiltration showed a significant negative correlation in multiple databases (Fig. 4B-E), and CD4 + T cell immune infiltration showed a significant positive correlation in TIMER (r(COAD) = 0.232, p(COAD) = 1.03e − 04; r(READ) = 0.284, p(READ) = 6.73e − 03)(Fig. 4F, G). In particular, ZNF880 expression and macrophage immune infiltration in 10 Significant positive correlation (Fig. 4H-Q) is shown in the database, and the correlation is strongest in TIMER (*r* = 0.323, *p* = 4.33e − 08). In addition, ZNF880 is also significantly related to the immune infiltration of neutrophils in COAD (Fig. 4R, S). These data indicate that ZNF880 may play a specific role in the immune infiltration of CRC.

### Correlation analysis between ZNF880 and immune cell marker genes

**Fig. 5:**
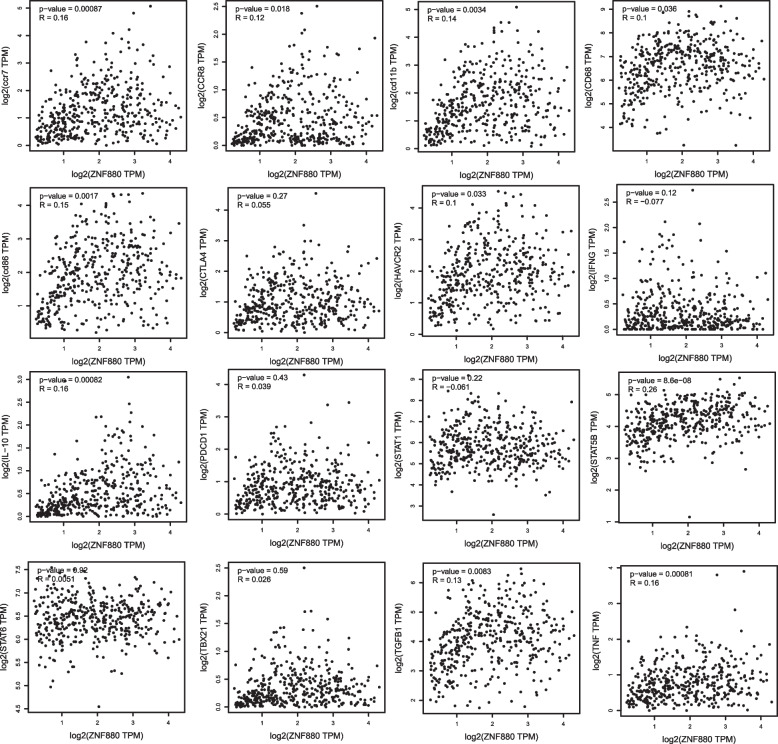
Correlation analysis between ZNF880 and 16 immune cell marker genes. Among them, 10 genes showed a significant positive correlation with ZNF880

To further determine the role of ZNF880 in tumor immunity, we analyzed the correlation between ZNF880 and 16 immune cell marker genes. The results show that ZNF880 has a significant correlation with 10 genes and a positive correlation with all 10 genes. These genes include CCR7 (*r* = 0.16, *p* = 0.0087), CCR8 (*r* = 0.12, *p* = 0.018), CD11B (*r* = 0.14, *p* = 0.0034), CD68 (*r* = 0.1, *p* = 0.036), CD86(*r* = 0.15, *p* = 0.0017), HAVCR2(*r* = 0.1, *p* = 0.033), IL-10(*r* = 0.16, *p* = 0.00082), STAT5B (*r* = 0.26, *p* = 8.6e − 08), TGFB1(*r* = 0.13, *p* = 0.0083), TNF (*r* = 0.16, *p* = 0.00081) (Fig. 5). In general, the decreased expression of ZNF880 may lead to a decrease in the level of immune infiltration of immune cells in CRC, which leads to a significant reduction in the function of immune cells in CRC.

### Low mRNA expression of ZNF880 is associated with short OS and DFS

**Fig. 6:**
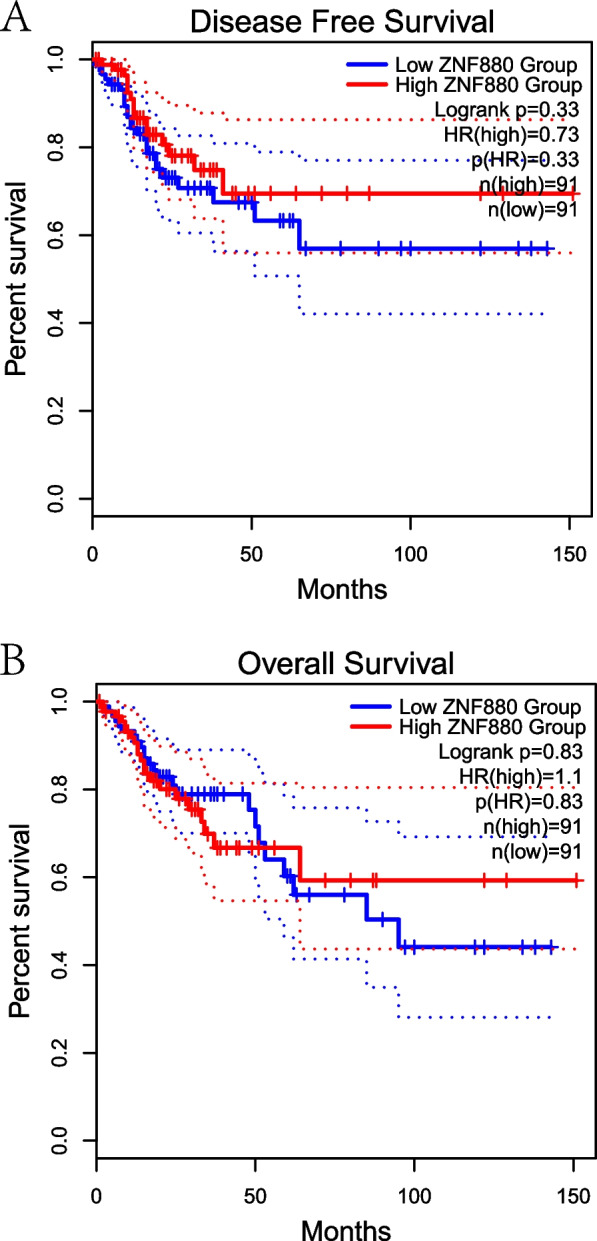
The prognosis of OS and DFS of ZNF880 in CRC patients

In order to explore the relationship between ZNF880 and OS and DFS in CRC patients, OS and DFS analysis based on the TCGA database was carried out. The results indicate that low expression of ZNF880 may lead to shorter DFS and OS (Fig. 6A, B). In order to further clarify the relationship between ZNF880 expression and the survival of CRC patients, we analyzed the CRC chip data set (GSE17536, GSE14333, GSE17537) to determine its correlation. The results showed that the expression of ZNF880 was significantly correlated with OS, DFS and DSS in CRC patients (Table .1). These data indicate that low mRNA expression of ZNF880 is a biomarker for poor prognosis of CRC.

**Table 1:** The correlation of ZNF880 with the prognosis of CRC patients in the GSE17536, GSE14333, and GSE17537 data sets

ID_NAME	DATASET	CANCER TYPE	ENDPOINT	COHORT	PROBE ID	*P*-VALUE
ZNF880	GSE17536	CRC	OS	MCC	232315_at	0.055368
ZNF880	GSE17536	CRC	DSS	MCC	232315_at	0.045468
ZNF880	GSE17536	CRC	OS	MCC	235913_at	0.015079
ZNF880	GSE17536	CRC	DSS	MCC	235913_at	0.007958
ZNF880	GSE17536	CRC	DFS	MCC	232315_at	0.002737
ZNF880	GSE17536	CRC	DFS	MCC	235913_at	0.03615
ZNF880	GSE14333	CRC	DFS	Melbourne	232315_at	0.023111
ZNF880	GSE14333	CRC	DFS	Melbourne	235913_at	0.022737
ZNF880	GSE17537	CRC	OS	VMC	232315_at	0.046462
ZNF880	GSE17537	CRC	OS	VMC	235913_at	0.037382
ZNF880	GSE17537	CRC	DFS	VMC	232315_at	0.005318
ZNF880	GSE17537	CRC	DFS	VMC	235913_at	0.018586
ZNF880	GSE17537	CRC	DSS	VMC	232315_at	0.037554
ZNF880	GSE17537	CRC	DSS	VMC	235913_at	0.020751

### PPI network construction and function prediction of ZNF880

**Fig. 7:**
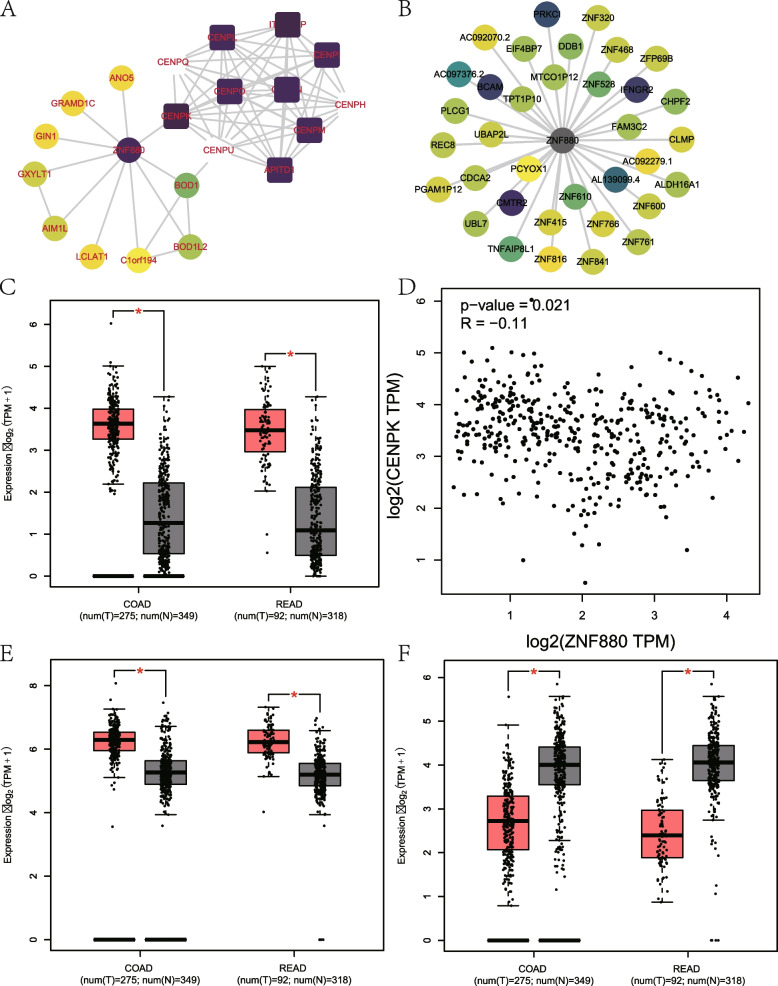
PPI network analysis of ZNF880 and difference analysis of key genes. **A**: The ZNF880 interaction network established by STRING. **B**: The gene network that ZNF880 may bind with predicted by homeopathy. **C**: The expression level of CENPK gene in READ and COAD. **D**: The correlation between CENPK gene and ZNF880 in CRC. **E**: The expression level of IFNGR2 gene in READ and COAD. **F**: The expression level of REC8 gene in READ and COAD

So far, there is no literature report on ZNF880 function analysis and disease-related. Therefore, we decided to use multiple databases to predict the functional pathways that ZNF880 may participate in. First, a ZNF880PPI network (Fig. 7A) with an interaction score > 0.4 was constructed from the STRING database. The results indicate that ZNF880 may interact with Interphase Centromere Complex Protein (CENPK). Furthermore, through the ENCORI database, it was constructed that ZNF880 may have homeopathic interactions with mRNA (Fig. 7B), and two key proteins, IFNGR2 and REC8, were screened by the free energy of binding between ZNF880 and them. Further analysis showed that CENPK and IFNGR2 showed up-regulated expression in READ and COAD (Fig. 7C, E), while REC8 showed down-regulated expression (Fig. 7F). In addition, ZNF880 and CENPK expression in CRC were significantly negatively correlated (*r* = -0.11, *p* = 0.021) (Fig. 7D). Finally, by analyzing the possible binding transcription factors of ZNF880 promoter and enhancer regions, it is found that ZBTB17 may be one of the key molecules regulating ZNF880. Similarly, ZBTB17 also showed down-regulation in READ and COAD, which may indicate that ZBTB17 is an agonist of ZNF880.

### Validation potential anti-cancer role of ZNF880

**Fig. 8:**
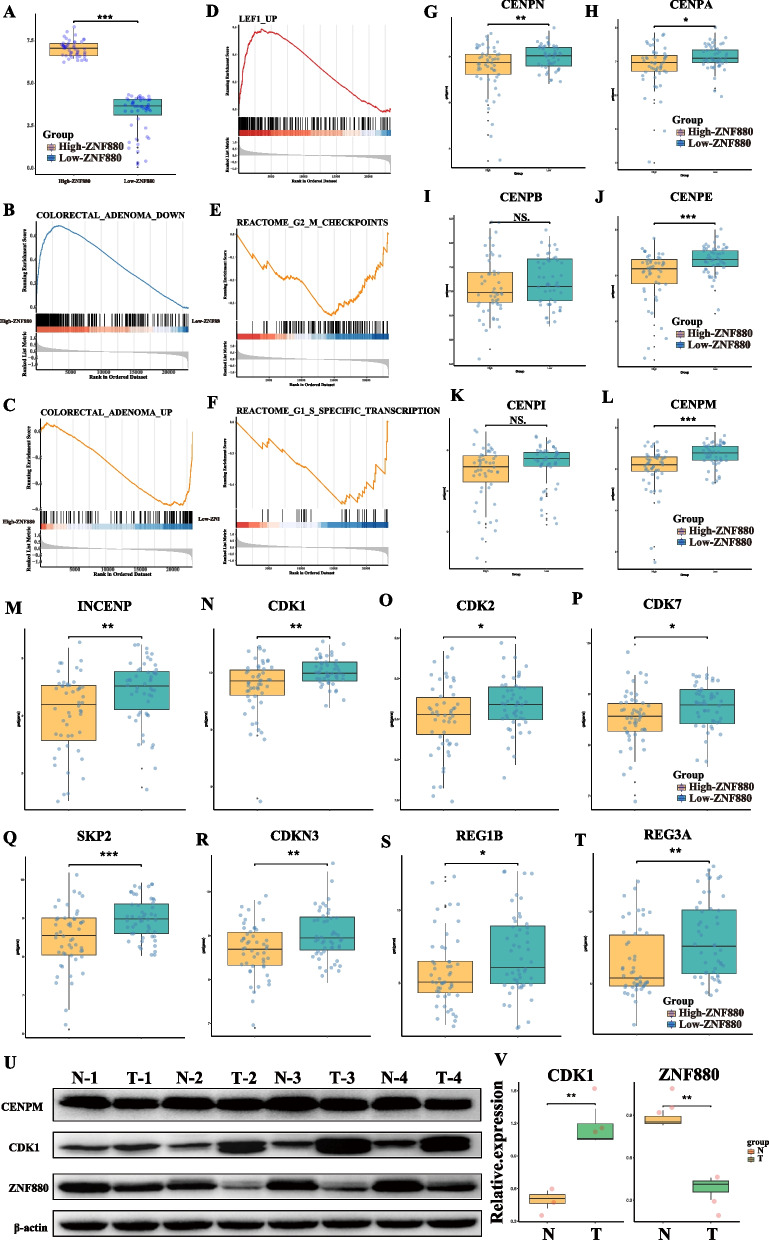
Individuals with high expression of ZNF880 and patients with low expression of ZNF880 in GSE14333 verify the potential function of ZNF880. **A**: Expression of ZNF880 between High-ZNF880 group and Low-ZNF880 group. **B**, **C**: GSEA analysis based on cell type signature gene sets, representing the enrichment of highly expressed genes in the High-ZNF880 group and the Low-ZNF880 group, respectively. **D**, **E**: GSEA analysis based on oncogenic signature gene sets. **G**-**M**: Differential expression of centriole protein between High-ZNF880 group and Low-ZNF880 group. **N**-**R**: Differential expression of cell cycle-dependent kinase genes and suppressor genes between High-ZNF880 group and Low-ZNF880 group. **S**, **T**: Differential expression of potential proto-oncogenes REG1B and REG3A between High-ZNF880 group and Low-ZNF880 group. **U**: Western blot results of ZNF880, CDK1, and CENPM in fresh tumor tissues and adjacent normal tissues from 4 matched patients with colorectal cancer (CRC), with β-actin as a reference. T-prefix represents tumor tissue, while N-prefix represents normal tissue. (Figs. S1, S2, S3, S4) **V**: Quantitative analysis of the Western blot band intensity of ZNF880 and CDK1 in 3 matched pairs of tumor tissues and adjacent normal tissues from colorectal cancer (CRC) patients with differential expression of ZNF880

The GSE14333 dataset was used as the validation cohort to further validate the hypothesis that ZNF880 may function as a possible tumor suppressor gene in CRC in this study. For comparison analysis, the 50 samples with the highest expression of ZNF880 and the 50 samples with the lowest expression of ZNF880 in GSE14333 were classified as the High-ZNF880 group and the Low-ZNF880 group, respectively (Fig. 8A). The differential gene analysis revealed 986 difference genes (*p* < 0.05, FC > 1.2 || FC 0.8) between High-ZNF880 and Low-ZNF880 patients. The GSEA analysis, which was based on the oncogenic signature gene sets dataset in the MSigDB database, revealed that the majority of the up-regulated genes in the High-ZNF880 group were enriched in the down-regulated gene sets in CRC, while the majority of the down-regulated genes in the High-ZNF880 group were enriched in the up-regulated genes in CRC. Sets of genes (Fig. 8B, C). This suggests that ZNF880 may decrease the expression of certain putative oncogenes while increasing the expression of tumor suppressor genes. Simultaneously, GSEA analysis of other data sets revealed that the up-regulated genes in the High-ZNF880 group were also significantly related to LEF1 UP (Fig. 8D), whereas the up-regulated genes in the Low-ZNF880 group were significantly related to G2 M CHECKPOINTS, G1 S SPECIFIC TRANSCRIPTION (Figs. 8E, F). LEF1 is a tumor suppressor gene found in CRC, suggesting that ZNF880 might be a possible positive regulator of LEF1. Simultaneously, ZNF880 may be a possible cell cycle regulator. To explore this theory further, we compared the alterations in critical genes associated to centriole and cell cycle regulation in the High-ZNF880 and Low-ZNF880 groups. In the Low-ZNF880 group, CENPN, CENPA, CENPE, CENPM, and INCENP were significantly increased (*p* < 0.05), while CENPB and CENPI were upregulated but not significantly (Fig. 8G-M). Simultaneously, CDK1, CDK2, CDK7, and SKP2, as well as other important kinase genes associated to cell cycle control, were considerably up-regulated in the Low-ZNF880 group (Fig. 8N-R). Furthermore, two possible proto-oncogenes, REG1B and REG3A, were shown to be up-regulated in the Low-ZNF880 group (Fig. 8S, T).

To further verify the reliability of our data, we collected fresh tumor tissues and adjacent normal tissues from 4 matched patients with colorectal cancer (CRC) from a hospital. We further validated our previously discovered patterns by Western blotting to determine their credibility. The results showed that ZNF880 was significantly downregulated at the protein level in the tumor tissues of 3 CRC patients (Fig. 8U, V). Further validation of CDK1 expression revealed that 3 ZNF880 low-expressing samples exhibited high protein levels (Fig. 8U, V, Fig. S1), while CENPM showed no significant change (Fig. 8U, V, Fig S2). These findings imply that ZNF880 may be a potential negative regulator of centriole organization and cell cycle regulation in CRC.

## Discussion

The KZNF gene family is highly complex and huge, and the functions of most of the KZNFs family members in cancer still need to be explored urgently. At the same time, increasing research gradually unravels the hypothetical function of KZNF family genes in various cancers [26–29]. KZNFs are known for their repressive function acting through epigenetic mechanisms, such as deposition of H3K9me3 and DNA methylation [18, 19]. It is worth noting that the genes under the control of KZNF may not only be suppressed, but also activated. Therefore, KZNFs play different functions in various types of cancer. The literature shows that some KZNFs are up-regulated in cancer, including ZNF695, ZNF320, ZNF200, ZNF354A, ZNF707, ZNF138 and so on [30–33]. Nevertheless, how these KZNFs affect tumor behavior is largely unknown. Therefore, more research is needed to further analyze and clarify the possible role of each cancer-related KZNFs in tumorigenesis. In this study, we determined the correlation between low expression of ZNF880 and the prognosis of CRC patients, and constructed and predicted the ZNF880 regulatory network and possible functional pathways. The results indicate that ZNF880 may exert its transcription factor activity by regulating downstream functional genes such as CENPK, IFNGR2, and REC8.

CENPK is component of the CENPA-CAD (nucleosome distal) complex, a complex recruited to centromeres which is involved in assembly of kinetochore proteins, mitotic progression and chromosome segregation [34–36]. CENPK may involved in incorporation of newly synthesized CENPA into centromeres via its interaction with the CENPA-NAC complex. In addition, CENPK can recruit the NDC80 complex to the external kinetochore through a synergistic effect with KNL1, which plays an important role in the pre-middle stage of mitosis [37]. Previous studies have shown that CENPK is abnormally up-regulated in a variety of tumor tissues and cells, and has been used as a new tumor marker in a variety of cancers such as hepatocellular carcinoma and ovarian cancer [35, 38–40]. Furthermore, CENPK knockdown significantly inhibited proliferation, migration, invasion, and EMT progression in HCC cells [35]. Further analysis showed that CENPK regulates the growth of tumor cells through YAP1 [35]. YAP1 is a key component of the Hippo signaling pathway, and has been shown to be essential for the initiation, progression, and metastasis of many types of cancer [41, 42]. In our analysis, ZNF880 was identified as interacting with CENPK. At the same time, CENPK and YAP1 were found to be up-regulated in CRC patients. We speculate that ZNF880 may be a repressor of CENPK gene expression, and the abnormal down-regulation of ZNF880 expression in CRC may lead to an increase in the expression of CENPK. The increase of CENPK expression further activates the expression of YAP1, which leads to the activation of CRC tumor signal pathway.

Bax is a major proapoptotic member of the B-cell lymphoma 2 (Bcl-2) family proteins that control apoptosis in normal and cancer cells [43–45]. Bax dysfunction can cause cancer cells to become resistant to treatment and promote tumorigenesis [46]. Previous studies have shown that the C-terminal cytoplasmic domain of IFNGR2 can bind to Bax and inhibit the conformational changes required for Bax activation, thereby inhibiting the function of Bax [47]. In the cis-acting target analysis of ZNF880, we identified that IFNGR2 may be one of the interacting proteins of ZNF880. The expression level of IFNGR2 in CRC is abnormally increased, far exceeding the gene IFNGR1 that is combined with it. This may indicate that IFNGR2 in CRC may not only bind to IFNGR1 to form a receptor for the cytokine interferon gamma (IFNG), but also may act alone. Plays an important role. We speculate that IFNGR2 may limit the activation of Bax protein conformation at the protein level. At the same time, ZNF880 may act as a transcriptional repressor of IFNGR2. The decreased expression of ZNF880 may lead to an abnormal increase in the level of IFNGR2, leading to the activation of the Bax protein conformation.

REC8 is a key meiosis-specific component of the cohesive complex and is related to DNA damage repair and chromosome stability maintenance [48]. REC8 has been shown to have tumor suppressor activity in a variety of cancers and is a new type of tumor suppressor gene [49–52]. Similarly, we found that REC8 was significantly down-regulated in CRC. In addition, the cis-acting site prediction of ZNF880 indicates that ZNF880 and REC8 may interact. This may indicate that ZNF880 may be an activator of REC8 transcription. The low expression of ZNF880 leads to a decrease in the expression of REC8 and thus limits the tumor suppressor activity of REC8.

In recent years, miRNAs have been extensively studied, but only a few lncRNAs have been studied in depth. In this study, we identified some specific lncRNA and miRNA directly related to ZNF880, and also provided a ceRNA network. The ceRNA network we constructed contains 112 lncRNAs, indicating that they play an important role in the development of CRC by regulating ZNF880 through competing miRNAs. The decreased expression of LINC00641, LINC00665, LINC01278, MALAT1, NEAT1, etc. in CRC may result in decreased binding to hsa-miR-126-5p, resulting in more hsa-miR-126-5p binding to ZNF880, leads to expression reduce of ZNF880. Competitively combining with hsa-miR-126-5p and other small RNAs that regulate the expression of ZNF880 through artificial intervention areas may be a new strategy to improve the prognosis of CRC patients and the treatment of CRC.

We have observed a concurrent downregulation of ZNF880 and upregulation of CDK1 in colorectal cancer samples, suggesting a potential direct or indirect regulatory relationship between the two. CDK1 is a critical cell cycle regulatory protein that plays a vital role at different stages of the cell cycle. It is closely associated with transitions in the cell cycle, including G1/S and G2/M transitions. CDK1 has been demonstrated to promote the proliferation, anti-apoptotic escape, as well as enhanced invasiveness and metastatic capacity of colorectal cancer cells. ZNF880 might directly target the promoter region of the CDK1 gene, influencing CDK1 expression by binding and modulating its transcriptional activity. Alternatively, ZNF880 could indirectly regulate CDK1 expression by modulating other transcription factors or molecules within signaling pathways. For instance, ZNF880 might be involved in regulating the upstream regulators, tumor suppressor genes, or cell cycle regulatory proteins of CDK1, thereby influencing its expression. In summary, ZNF880 may regulate CDK1 expression levels through transcriptional repression, thereby impacting the progression of colorectal cancer.

Despite the relationship between ZNF880 and CDK1 still being under preliminary investigation, these observations have already provided valuable directions for our future research. Next steps could involve gene knockdown or overexpression studies to explore whether ZNF880 influences the expression and activity of CDK1, thereby determining their relationship. Techniques such as immunoprecipitation and mass spectrometry could aid in determining whether ZNF880 and CDK1 directly interact, while also examining if ZNF880 regulates the transcription of the CDK1 gene. Functional studies using colorectal cancer models along with ChIP-seq to study the target genes and binding sites of ZNF880 will contribute to a comprehensive understanding of ZNF880's function and regulatory mechanism in colorectal cancer.

We have observed a concurrent downregulation of ZNF880 and upregulation of CDK1 in colorectal cancer samples, suggesting a potential direct or indirect regulatory relationship between the two. CDK1 is a critical cell cycle regulatory protein that plays a vital role at different stages of the cell cycle. It is closely associated with transitions in the cell cycle, including G1/S and G2/M transitions. CDK1 has been demonstrated to promote the proliferation, anti-apoptotic escape, as well as enhanced invasiveness and metastatic capacity of colorectal cancer cells. ZNF880 might directly target the promoter region of the CDK1 gene, influencing CDK1 expression by binding and modulating its transcriptional activity. Alternatively, ZNF880 could indirectly regulate CDK1 expression by modulating other transcription factors or molecules within signaling pathways. For instance, ZNF880 might be involved in regulating the upstream regulators, tumor suppressor genes, or cell cycle regulatory proteins of CDK1, thereby influencing its expression. In summary, ZNF880 may regulate CDK1 expression levels through transcriptional repression, thereby impacting the progression of colorectal cancer.

Despite the relationship between ZNF880 and CDK1 still being under preliminary investigation, these observations have already provided valuable directions for our future research. Next steps could involve gene knockdown or overexpression studies to explore whether ZNF880 influences the expression and activity of CDK1, thereby determining their relationship. Techniques such as immunoprecipitation and mass spectrometry could aid in determining whether ZNF880 and CDK1 directly interact, while also examining if ZNF880 regulates the transcription of the CDK1 gene. Functional studies using colorectal cancer models along with ChIP-seq to study the target genes and binding sites of ZNF880 will contribute to a comprehensive understanding of ZNF880's function and regulatory mechanism in colorectal cancer.

## Supplementary Information

**Additional file 1. Figure S1. **The original Western blot band of CDK1.

**Additional file 2. Figure S2. **The original Western blot band of CENPM.

**Additional file 3. Figure S3. **The original Western blot band of ZNF880.

**Additional file 4. Figure S4. **The original Western blot band of β-actin.

**Additional file 5. Table S1.** Interaction prediction between candidate miRNAs and lncRNAs.

## Data Availability

The datasets analysed during the current study are publicly available in TCGA (https://www.genome.gov/Funded-Programs-Projects/Cancer-Genome-Atlas) and UALCAN (http:/ /ualcan.path.uab.edu/analysis.html). The GEO data used in this study were downloaded from NCBI via GSE17536, GSE14333, GSE17537.

## Abbreviation

KRAB: Krüppel-associated box
KZNF: KRAB-type ZNF transcription factor family
OS: Overall survival
DFS: Disease-free survival
CRC: Colorectal carcinoma
COAD: Colon adenocarcinoma
READ: Rectum adenocarcinoma
SNV: Single nucleotide variation
