# Supplementary figures and images for: Promoter hypermethylation and comprehensive regulation of ncRNA lead to the down-regulation of ZNF880, providing a new insight for the therapeutics and research of colorectal cancer
Source: BMC Med Genomics. 2023 Jun 27;16:148. doi: 10.1186/s12920-023-01571-2 (PMC10294494; doi:10.1186/s12920-023-01571-2)

**CDK1**

**
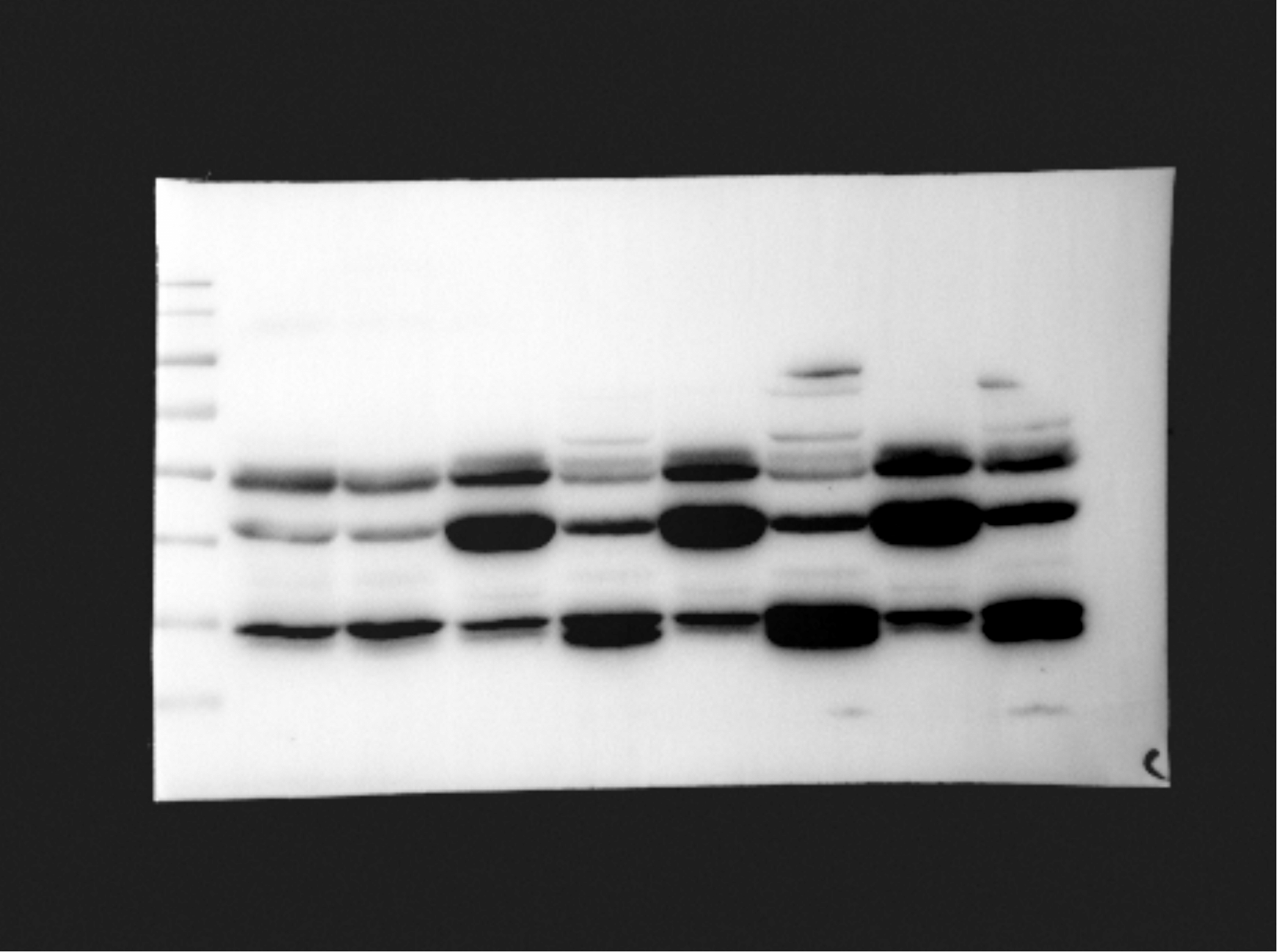
**

Supplement: Supplementary file 1 — Additional file 1. Figure S1. The original Western blot band of CDK1. [file 12920_2023_1571_MOESM1_ESM.docx]

**CENPM**

**
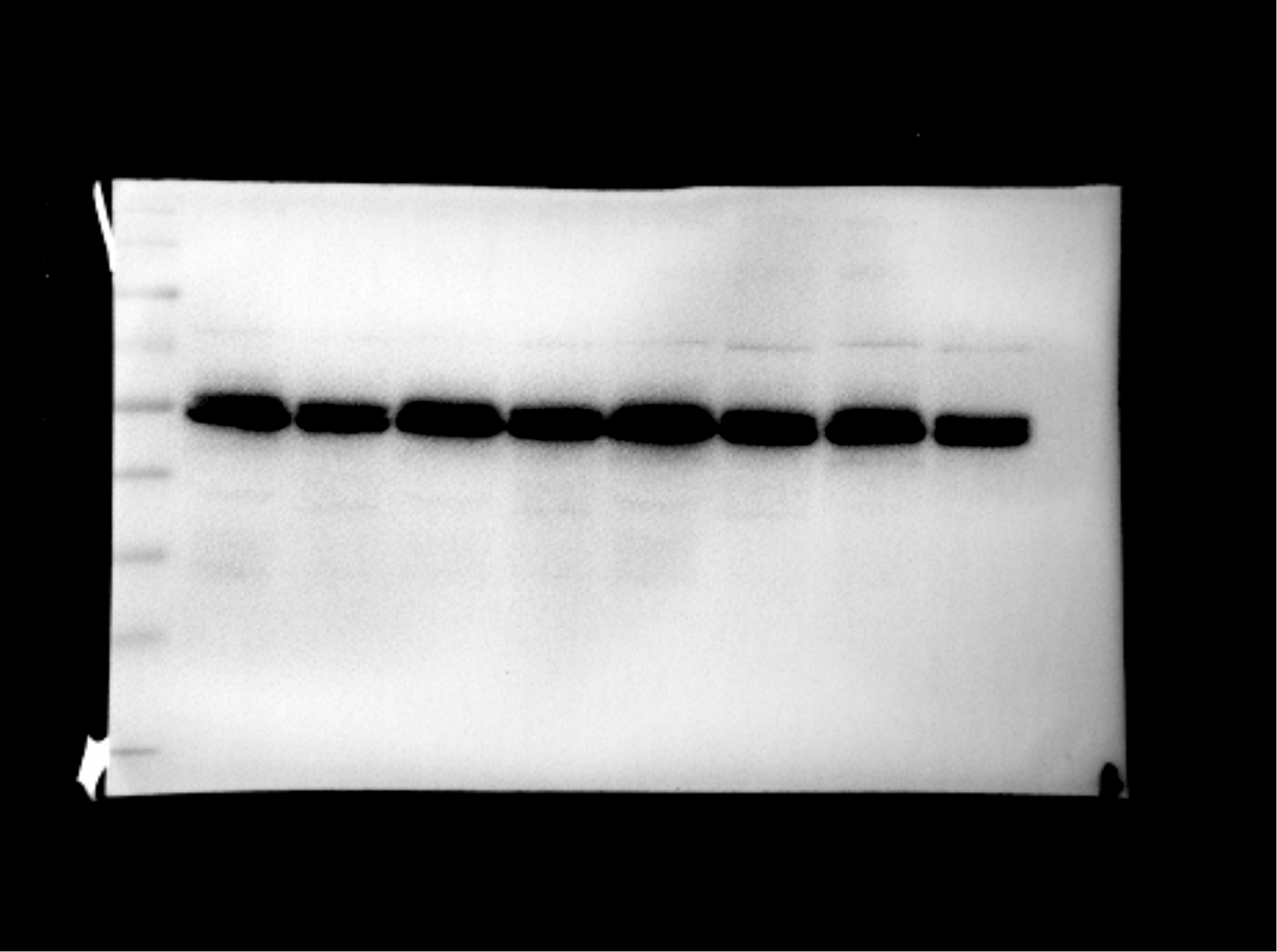
**

Supplement: Supplementary file 2 — Additional file 2. Figure S2. The original Western blot band of CENPM. [file 12920_2023_1571_MOESM2_ESM.docx]

**ZNF880**

**
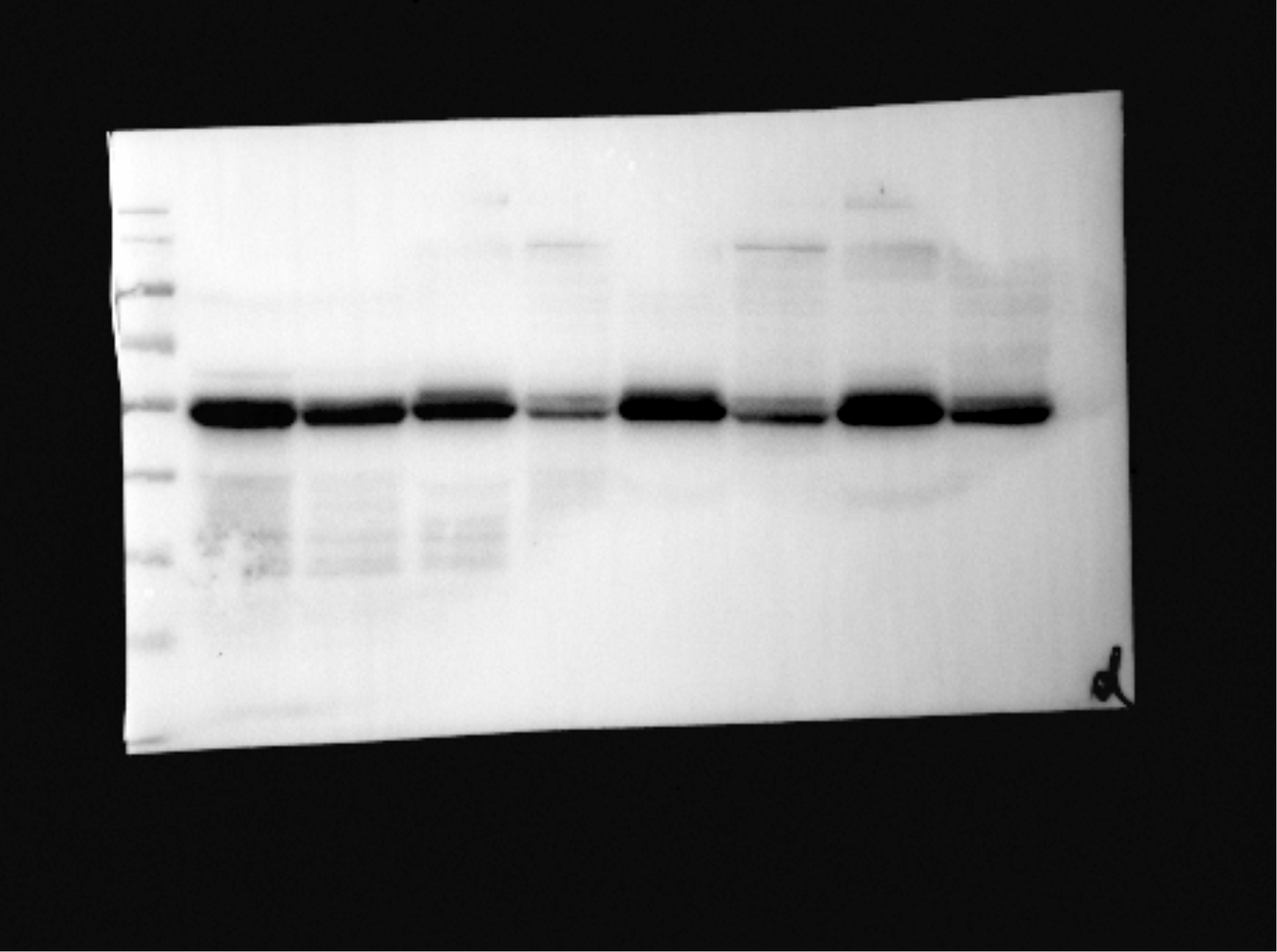
**

Supplement: Supplementary file 3 — Additional file 3. Figure S3. The original Western blot band of ZNF880. [file 12920_2023_1571_MOESM3_ESM.docx]

**β-actin**

**
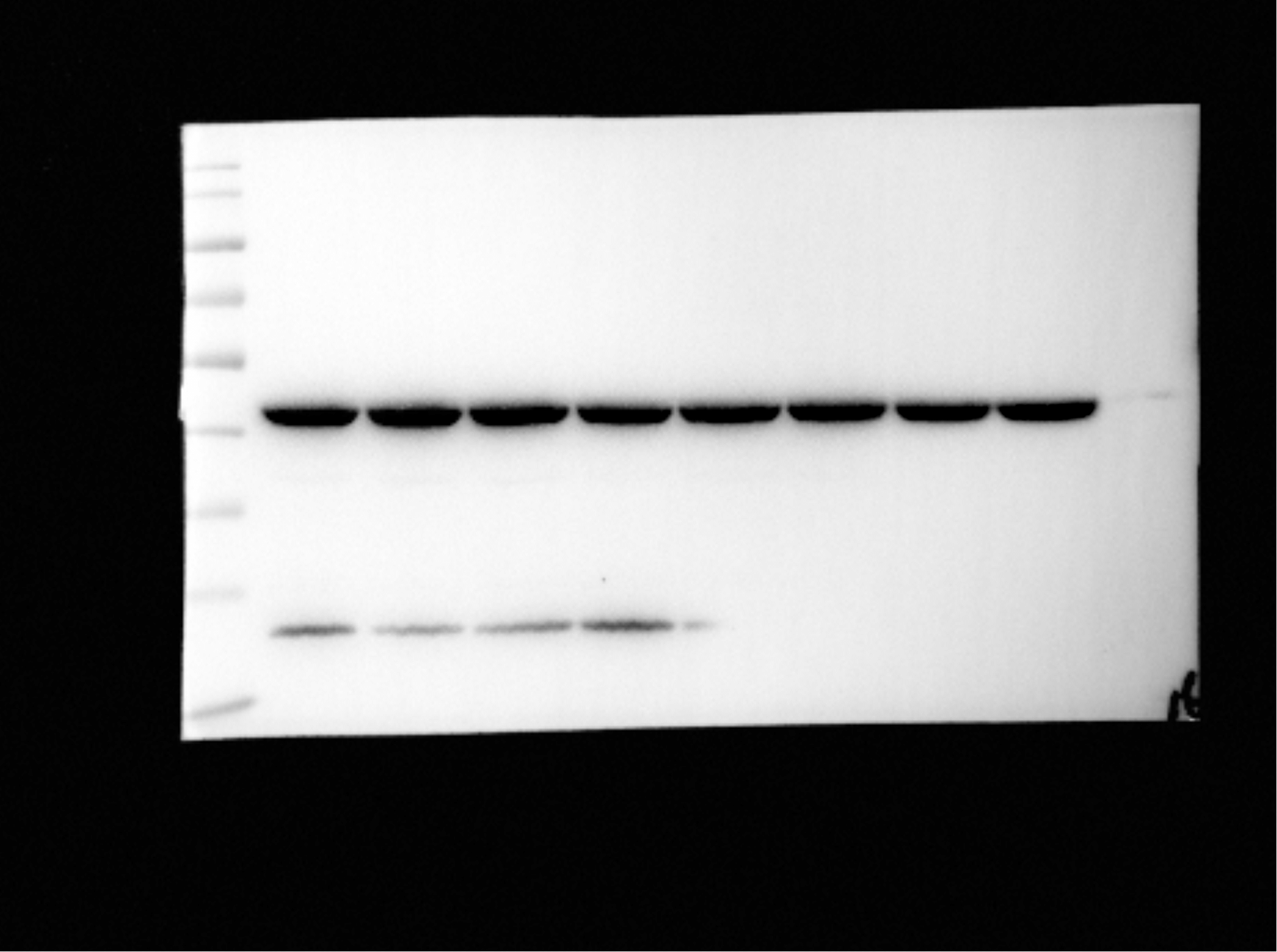
**

Supplement: Supplementary file 4 — Additional file 4. Figure S4. The original Western blot band of β-actin. [file 12920_2023_1571_MOESM4_ESM.docx]
